# Associations Between Anti-TNF Pharmacokinetics, Immunogenicity, and Therapeutic Response in Iraqi Patients with Inflammatory Bowel Disease: A Real-World Therapeutic Drug Monitoring Study

**DOI:** 10.3390/diseases14090325

**Published:** 2026-09-05

**Authors:** Ban AbdulWahid Kanna, Suha Saeed Azeez

**Affiliations:** Clinical Pharmacy Department, College of Pharmacy, Hawler Medical University, Erbil 44001, Iraq; ban.kanna@yahoo.com

**Keywords:** inflammatory bowel disease, infliximab, adalimumab, therapeutic drug monitoring, immunogenicity

## Abstract

**Background**: Therapeutic drug monitoring (TDM) has emerged as an important strategy for optimizing anti-tumor necrosis factor (anti-TNF) therapy in patients with inflammatory bowel disease (IBD). However, data regarding anti-TNF pharmacokinetics and immunogenicity from Middle Eastern populations remain limited. This study evaluated the associations between serum anti-TNF trough concentrations, anti-drug antibody (ADAb) levels, and therapeutic response among Iraqi patients with IBD receiving infliximab or adalimumab therapy. **Methods**: This cross-sectional observational study included 80 patients with Crohn’s disease or ulcerative colitis receiving maintenance infliximab or adalimumab therapy at a tertiary Gastroenterology Center in Iraq. Patients were categorized as responders or non-responders according to clinical disease activity indices and biochemical assessment. Serum trough levels of infliximab and adalimumab, as well as ADAb concentrations, were measured using an enzyme-linked immunosorbent assay (ELISA). **Results**: Responders had significantly higher serum trough concentrations than non-responders for both infliximab [3.75 µg/mL (IQR: 3.40–4.15) vs. 1.05 µg/mL (IQR: 0.92–1.12), *p* < 0.001] and adalimumab [6.32 µg/mL (IQR: 5.67–7.15) vs. 3.30 µg/mL (IQR: 2.70–4.26), *p* < 0.001]. Adalimumab-treated non-responders had significantly higher ADAb levels compared with responders (*p* = 0.047). **Conclusions**: Favorable therapeutic outcomes in Iraqi IBD patients treated with anti-TNF agents were associated with adequate serum trough levels and low ADAb levels. Reactive therapeutic drug monitoring may provide clinically valuable pharmacokinetic information capable of guiding individualized treatment optimization and identifying mechanisms of treatment failure in patients receiving infliximab or adalimumab therapy.

## 1. Introduction

Inflammatory bowel disease (IBD), primarily comprising Crohn’s disease (CD) and ulcerative colitis (UC), is a chronic, immune-mediated disorder of the gastrointestinal tract characterized by a relapsing and remitting course. Globally, the incidence and prevalence of IBD are rising, posing significant public health challenges [1]. Emerging epidemiological evidence from Iraq and neighboring Middle Eastern countries also suggests an increasing regional burden of disease, particularly among younger populations during their most productive years [2,3]. Over the past two decades, the introduction of anti-TNF agents has revolutionized the therapeutic landscape of moderate-to-severe IBD. Agents such as infliximab (IFX) and adalimumab (ADM) have become cornerstones of medical therapy, enabling patients to achieve high rates of clinical remission, durable mucosal healing, and a significantly reduced need for surgical interventions [4,5]. Despite the effectiveness of these biologics in treating IBD, about one third (10–30%) of patients do not respond to anti-TNF agents during the induction phase, a phenomenon known as primary non-response (PNR), whereas 23–46% of patients lose response during treatment, a phenomenon referred to as secondary loss of response (LOR) [6,7]. This LOR is largely driven by altered pharmacokinetics and immunogenicity. Inter-individual variability in drug clearance can lead to subtherapeutic drug concentrations, which heavily predisposes patients to the formation of anti-drug antibodies (ADAbs). The development of ADAbs further accelerates drug clearance and neutralizes the medication’s therapeutic effect, resulting in clinical flares [8,9].

To address the challenges of primary non-response and secondary loss of response (LOR), therapeutic drug monitoring (TDM) has emerged as a pivotal clinical tool. TDM involves measuring biologic serum trough concentrations and anti-drug antibody (ADAb) levels to guide personalized dosing and optimize therapeutic outcomes. Treatment failure can occur in one of three patterns: mechanistic failure, where patients have optimal drug trough levels but show no response, likely linked to disease processes caused by inflammatory mediators not inhibited by the specific drug; non-immune-mediated pharmacokinetic failure, which occurs when the trough serum level is subtherapeutic and ADAbs are absent; and immune-mediated pharmacokinetic failure, which occurs when patients have high ADAb levels and low or undetectable serum trough levels due to the formation of neutralizing ADAbs that block the drug’s effect [10,11]. Historically, TDM has been utilized reactively, assessing drug and antibody levels only when a patient experiences active symptoms or loss of clinical response. Reactive TDM has proven highly effective in identifying the underlying cause of LOR and guiding rational therapeutic adjustments, such as dose escalation, switching within the same class, or switching to a biologic with a different mechanism of action [10].

Despite growing evidence, the relationship between pharmacokinetics, immunogenicity, and treatment response remains incompletely defined in different patient populations. Therefore, this study aimed to evaluate the associations between serum anti-TNF trough levels, ADAb formation, and therapeutic outcomes in patients with IBD.

## 2. Materials and Methods

### 2.1. Study Design and Setting

This single-center cross-sectional observational study was conducted at a tertiary referral Gastroenterology Center in Erbil, Kurdistan Region of Iraq, between October 2024 and August 2025. The study investigated how serum trough levels of anti-TNF agents, ADAb development, and clinical response are related in patients with IBD undergoing maintenance therapy with infliximab or adalimumab.

The study protocol was approved by the institutional scientific and ethical review committee of the College of Pharmacy, Hawler Medical University, Reference No.: HMU-EC-Ph 23102024-156, date: 23 October 2024. The sample size was calculated using the following formula: Sample size = z^2^ × p(1 − p)/d^2^. In this expression, z is the standard normal deviate corresponding to the chosen Type I error (for a two-sided test, z = 1.96 for *p* < 0.05, and z = 2.58 for *p* < 0.01). The variable *p* is the expected proportion in the population, estimated from previous studies or a pilot study. The parameter d represents the desired absolute precision (margin of error). All procedures were conducted in accordance with the ethical principles of the Declaration of Helsinki and its subsequent amendments [12]. Written informed consent was obtained from all participants before enrollment. Furthermore, the study was conducted and reported according to the Strengthening the Reporting of Observational Studies in Epidemiology (STROBE) recommendations for observational studies [13].

### 2.2. Study Population

A total of 80 adult patients with confirmed Crohn’s disease (CD) or ulcerative colitis (UC) were consecutively recruited during routine outpatient follow-up visits. The diagnosis of IBD was established according to standard clinical, endoscopic, radiologic, and histopathologic criteria consistent with contemporary international guidelines [14,15].

Eligible patients were 18 years or older and had confirmed CD or UC. Patients were eligible if they had completed the 12-week induction phase of infliximab (IFX) or adalimumab (ADM), transitioned to scheduled maintenance therapy, and received a total of 16 weeks or more of treatment prior to enrollment. Patients receiving induction anti-TNF therapy, those with active severe infection or malignancy, pregnant or lactating women, patients with incomplete clinical data, and individuals receiving biologic agents other than IFX or ADM were excluded from the study.

All patients received anti-TNF therapy according to standard induction and maintenance regimens. IFX was administered intravenously at 5 mg/kg at weeks 0, 2, and 6 for induction, followed by 5 mg/kg every 8 weeks for maintenance. ADM was given subcutaneously as 160 mg at week 0, 80 mg at week 2, then 40 mg every other week from week 4 onward. For some patients in both the IFX- and ADM-treated groups, the dosing interval was shortened based on the gastroenterologist’s decision. Patients in this cohort were divided into three groups according to therapy duration (<12 months, 12–24 months, and >24 months).

### 2.3. Clinical Assessment and Therapeutic Response

Demographic and clinical characteristics were collected from patient interviews and medical records, including age, sex, body mass index (BMI), smoking status, disease duration, previous anti-TNF exposure, concomitant azathioprine use, dosing regimen, and duration of biologic therapy.

Clinical disease activity was assessed at the time of blood sampling. Crohn’s disease activity was evaluated using the Harvey–Bradshaw Index (HBI), and ulcerative colitis activity was assessed using the Simple Clinical Colitis Activity Index (SCCAI) [16,17].

Patients were classified as responders or non-responders according to clinical disease activity indices and biochemical assessment [18,19]. Clinical remission (responders) was defined using the remission score (HBI ≤ 4 for CD, SCCAI ≤ 2 for UC) according to disease activity indices with CRP < 5 mg/L. Active disease/non-response was defined as mild or persistent moderate-to-severe disease activity score (HBI ≥ 5 for CD, SCCAI ≥ 3 for UC) with CRP ≥ 5 mg/L, corticosteroid requirement, or treatment optimization.

### 2.4. Blood Sampling and Laboratory Analysis

Peripheral venous blood samples were collected immediately before the next scheduled anti-TNF dose to measure trough concentration. Samples were centrifuged, and serum aliquots were stored at −80 °C until analysis. Serum trough concentrations of infliximab and adalimumab, as well as corresponding ADAb levels, were quantified using commercially available sandwich enzyme-linked immunosorbent assay (ELISA) kits according to the manufacturer’s instructions. The sandwich ELISA kits were manufactured by (RayBiotech Life, Inc., Peachtree Corners, GA, USA), including inflixiamb (Catalog No. EAD-Infl, LOT 01082692, sensitivity 0.002 ng/mL), anti-infliximab (Catalog No. EAD-AnInfl, LOT 01082692, sensitivity 0.007 ng/mL), adalimumab (Catalog No. EAD-Adal, LOT 01082692, sensitivity 0.012 ng/mL), and anti-adalimumab (Catalog No. EAD-AnAdal, LOT 01082692, sensitivity 0.25 ng/mL).

At the time of measurement, biologic serum trough levels and ADAb concentrations were determined in parallel from the same stored serum samples using manufacturer-defined ELISA kits. All assays were performed according to the respective kit instructions, with samples processed in a single batch to minimize inter-assay variability.

Therapeutic trough achievement was defined using clinically accepted maintenance targets of ≥3 µg/mL for infliximab and ≥5 µg/mL for adalimumab, consistent with contemporary TDM recommendations and previous pharmacokinetic studies [20,21].

### 2.5. Pharmacokinetic–Immunogenic Classification

Patients were additionally categorized according to combined pharmacokinetic and immunogenic profiles into therapeutic trough/low ADAb, therapeutic trough/high ADAb, subtherapeutic trough/low ADAb, and subtherapeutic trough/high ADAb groups. This classification was performed to evaluate patterns of pharmacokinetic and immune-mediated therapeutic failure consistent with current therapeutic drug monitoring algorithms [22,23].

### 2.6. Statistical Analysis

Statistical analysis was performed using IBM SPSS Statistics version 26.0 (IBM Corp., Armonk, NY, USA) and Python statistical packages (Python 3.x, Version 5).

Continuous variables were assessed for normality using the Shapiro–Wilk test. Normally distributed variables were presented as mean ± standard deviation (SD), whereas non-normally distributed variables were expressed as median and interquartile range (IQR). Categorical variables were summarized as frequencies and percentages. Comparisons between responders and non-responders were performed using Student’s t-test for normally distributed continuous variables, the Mann–Whitney U test for non-normally distributed variables, and the Chi-square test or Fisher’s exact test for categorical variables, as appropriate.

Spearman correlation analysis was used to evaluate associations between serum trough concentrations and ADAb levels. Receiver operating characteristic (ROC) curve analysis was performed to determine the discriminative ability and optimal cutoff values of trough concentrations and ADAb levels for predicting therapeutic response. The area under the curve (AUC), sensitivity, and specificity were calculated using the trapezoidal rule (nonparametric method).

Multivariable logistic regression analysis was performed to evaluate independent factors associated with therapeutic response after adjustment for clinically relevant covariates. Because partial and quasi-complete separation were observed during modeling, penalized logistic regression analysis was also performed using Firth’s approach to obtain more stable parameter estimates and reduce small-sample bias.

Sensitivity analyses were conducted after conservative winsorization of biologically extreme ADAb values to evaluate the robustness of the correlations. A two-sided *p*-value < 0.05 was considered statistically significant.

## 3. Results

### 3.1. Patients’ Baseline Demographic and Clinical Characteristics

**Figure 1:**
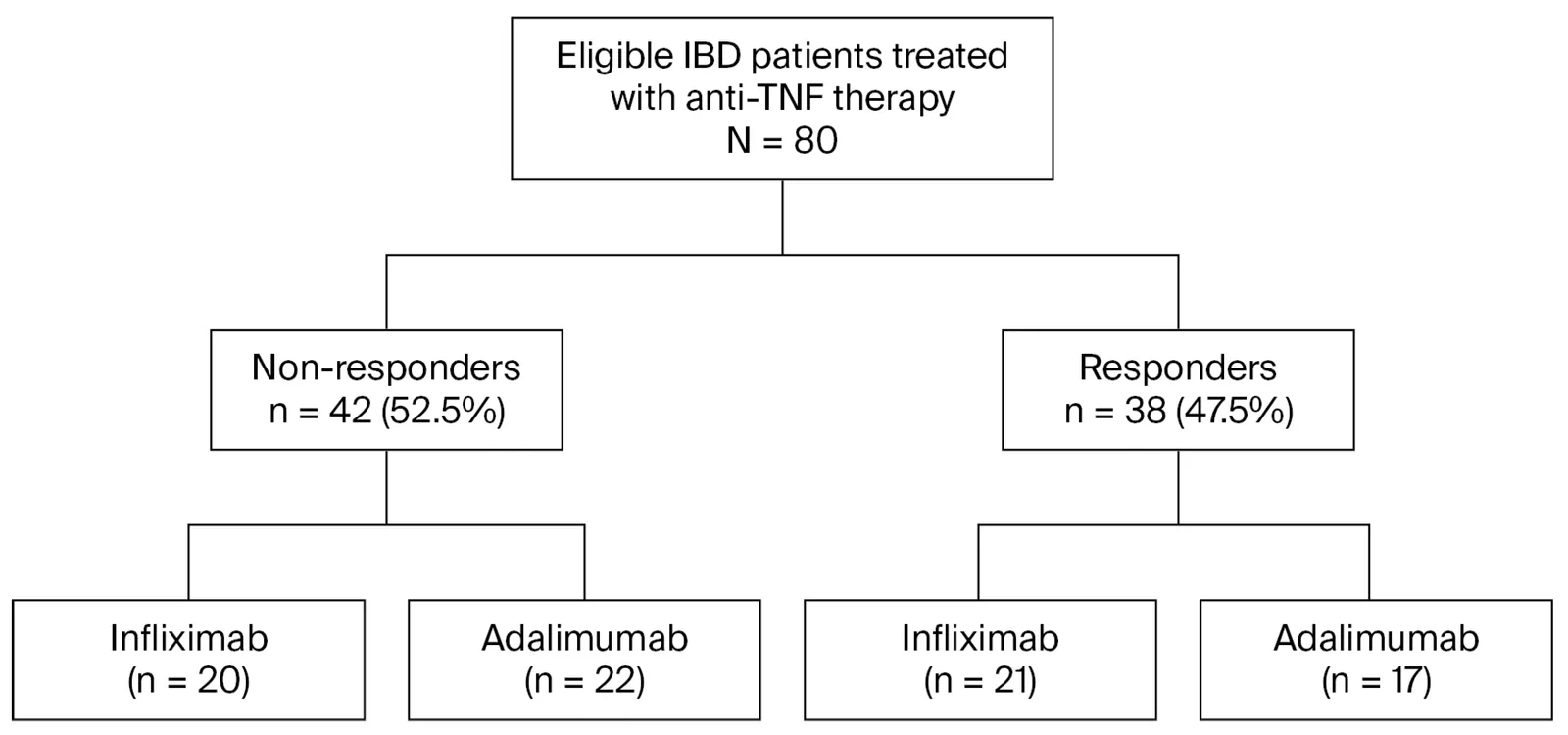
Anti-TNF-treated IBD patient selection and therapeutic response distribution.

A total of 80 patients with inflammatory bowel disease were recruited in the study, including patients with Crohn’s disease (*n* = 50) and ulcerative colitis (*n* = 30) receiving maintenance infliximab or adalimumab therapy, of whom 42 were male and 38 were female. Forty-one patients received infliximab and 39 received adalimumab. Within the infliximab group, 22 patients received concomitant azathioprine (combination therapy) and 19 received infliximab as monotherapy. In the adalimumab group, 13 patients received combination therapy and 26 received monotherapy. Standard dosing schedules were used in 55 patients, while 25 underwent dose intensification (shortened dosing interval). Fifteen patients had prior exposure to anti-tumor necrosis factor (anti-TNF) agents, sixty-five were anti-TNF-naïve, and four patients were receiving concomitant corticosteroid therapy. Overall, 38 patients (47.5%) were classified as responders, whereas 42 patients (52.5%) were classified as non-responders. The study flowchart and patient allocation are illustrated in Figure 1.

Baseline demographic and clinical characteristics according to therapeutic response are summarized in Table 1. No significant differences were observed between responders and non-responders regarding age, sex, body mass index, smoking status, disease duration, disease subtype, previous anti-TNF exposure, concomitant azathioprine use, or therapy duration (all *p* > 0.05). The only significant associations detected were with disease activity indices. In CD patients, responders showed a significantly lower median HBI score than non-responders [2.5 scores (IQR: 1–3) vs. 6 scores (IQR: 5–9.25), *p* < 0.001]. Likewise, in UC patients, responders showed significantly lower median SCCAI scores than non-responders [1 score (IQR: 0.25–1.75) vs. 4 scores (IQR: 3.75–5.25), *p* < 0.001].

### 3.2. Association Between Serum Anti-TNF Trough Concentrations and Therapeutic Response

**Figure 2:**
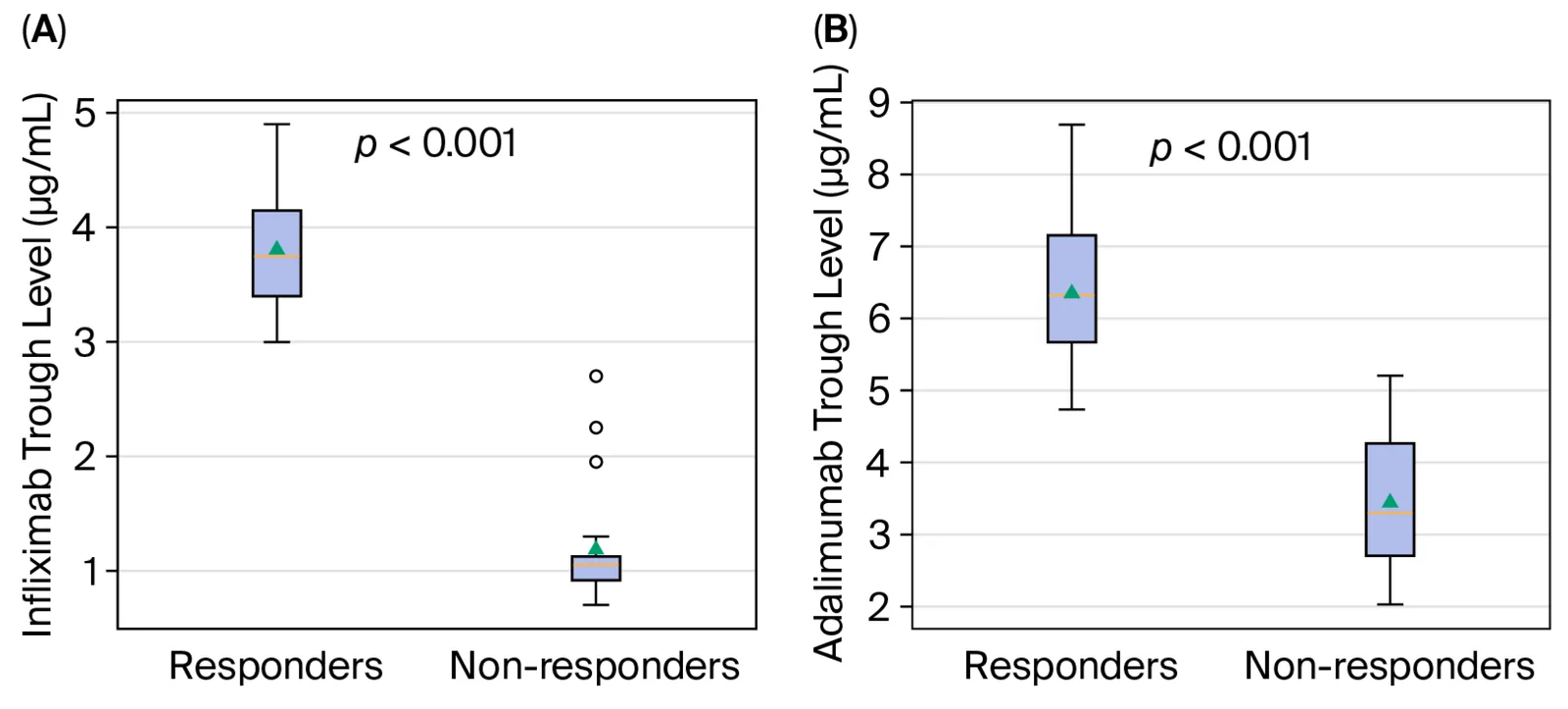
Trough concentrations according to therapeutic response. (**A**) Infliximab trough concentrations were significantly higher in responders compared to non-responders. (**B**) Adalimumab trough concentrations were significantly higher in responders compared to non-responders.

Responders had significantly higher serum anti-TNF trough concentrations compared with non-responders in both treatment groups. Among infliximab-treated patients, responders had significantly higher trough concentrations than non-responders [3.75 µg/mL (IQR: 3.40–4.15) vs. 1.05 µg/mL (IQR: 0.92–1.12), *p* < 0.001]. Similarly, among adalimumab-treated patients, responders had significantly higher trough concentrations than non-responders [6.32 µg/mL (IQR: 5.67–7.15) vs. 3.30 µg/mL (IQR: 2.70–4.26), *p* < 0.001]. These differences are illustrated in Figure 2A,B.

### 3.3. Receiver Operating Characteristic (ROC) Curve Analysis

ROC curve analysis demonstrated strong discriminative performance of serum anti-TNF trough concentrations for predicting therapeutic response.

**Figure 3:**
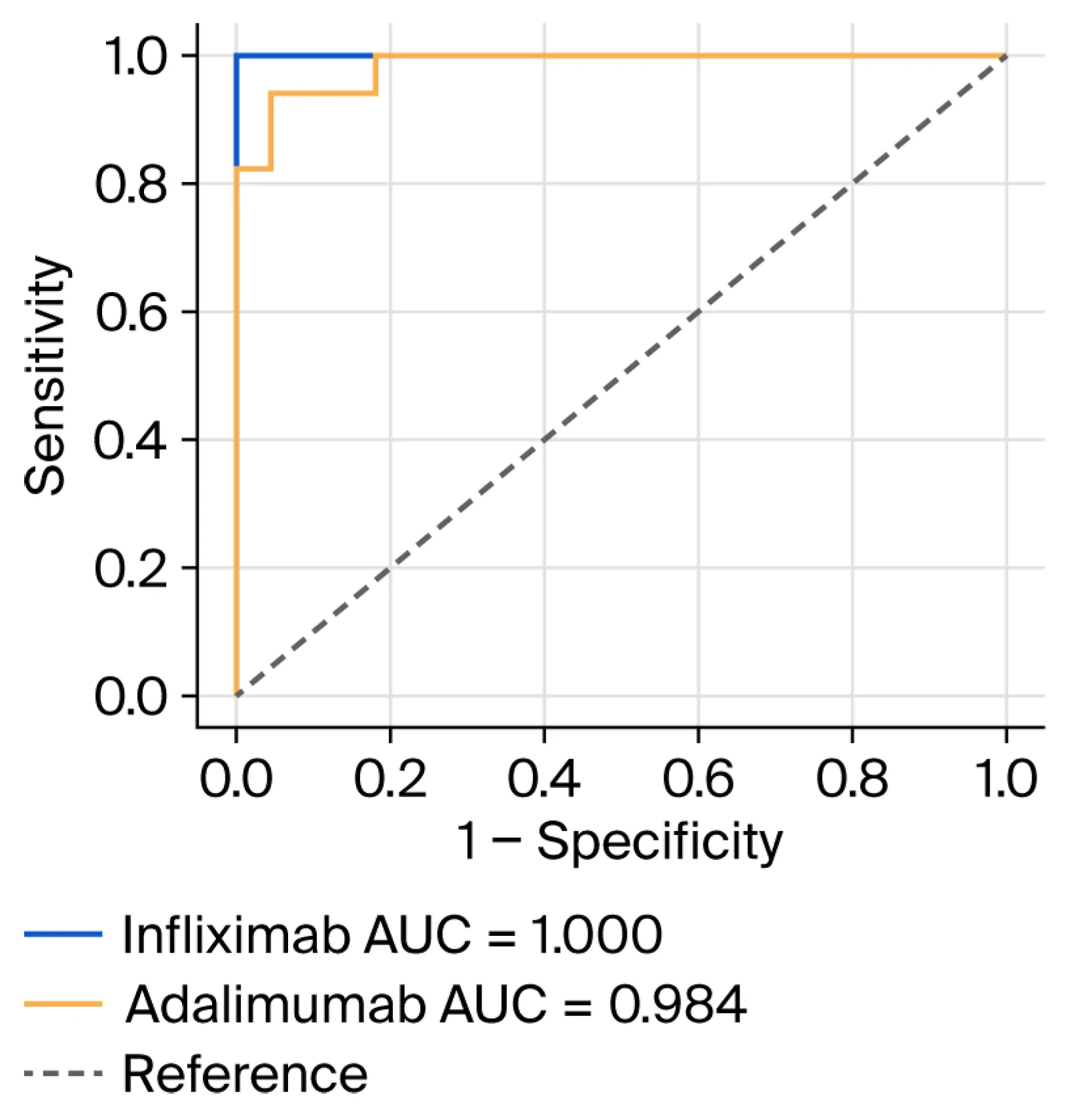
Receiver operating characteristic (ROC) curves showing the discriminative ability of anti-TNF trough concentrations to predict therapeutic response in infliximab- and adalimumab-treated patients.

For infliximab-treated patients, the optimal trough concentration cutoff for predicting response was 3.0 µg/mL, with an area under the curve (AUC) of 1.000, sensitivity of 100%, and specificity of 100%. For adalimumab-treated patients, the optimal trough concentration cutoff was 4.98 µg/mL, with an AUC of 0.984, sensitivity of 93.8%, and specificity of 94.1%. ROC curve analyses are presented in Figure 3. Although the discriminative performance observed in the present cohort was excellent, particularly for infliximab, the near-perfect ROC performance should be interpreted cautiously because the relatively modest sample size and partial cohort separation may have contributed to optimistic performance estimates.

### 3.4. Multivariate Logistic Regression Analysis

Multivariate logistic regression analysis was performed for infliximab and adalimumab to evaluate factors independently associated with therapeutic response after adjustment for clinically relevant covariates, including trough concentrations, ADAb levels, disease type, previous anti-TNF exposure, and concomitant azathioprine use.

Higher serum trough concentrations remained independently associated with therapeutic response after multivariable adjustment. In contrast, elevated ADAb levels demonstrated no statistically significant independent association after adjustment. Detailed regression results are summarized in Table 2 and Table 3.

### 3.5. Association Between Anti-Drug Antibody Levels and Therapeutic Response

Anti-drug antibody levels differed significantly according to therapeutic response, particularly in adalimumab-treated patients.

In the infliximab group, non-responders demonstrated higher ADAb concentrations than responders; however, this difference did not reach statistical significance. In contrast, adalimumab-treated non-responders had significantly higher ADAb levels compared with responders [61.35 ng/mL (IQR: 48.75–84.83) vs. 48.30 ng/mL (IQR: 35.70–59.70), *p* = 0.047]. Detailed ADAb comparisons according to treatment response are summarized in Table 4.

### 3.6. Correlation Between Serum Trough Concentrations and ADAb Levels

Spearman correlation analysis demonstrated strong inverse associations between serum anti-TNF trough concentrations and ADAb levels in both treatment groups.

**Figure 4:**
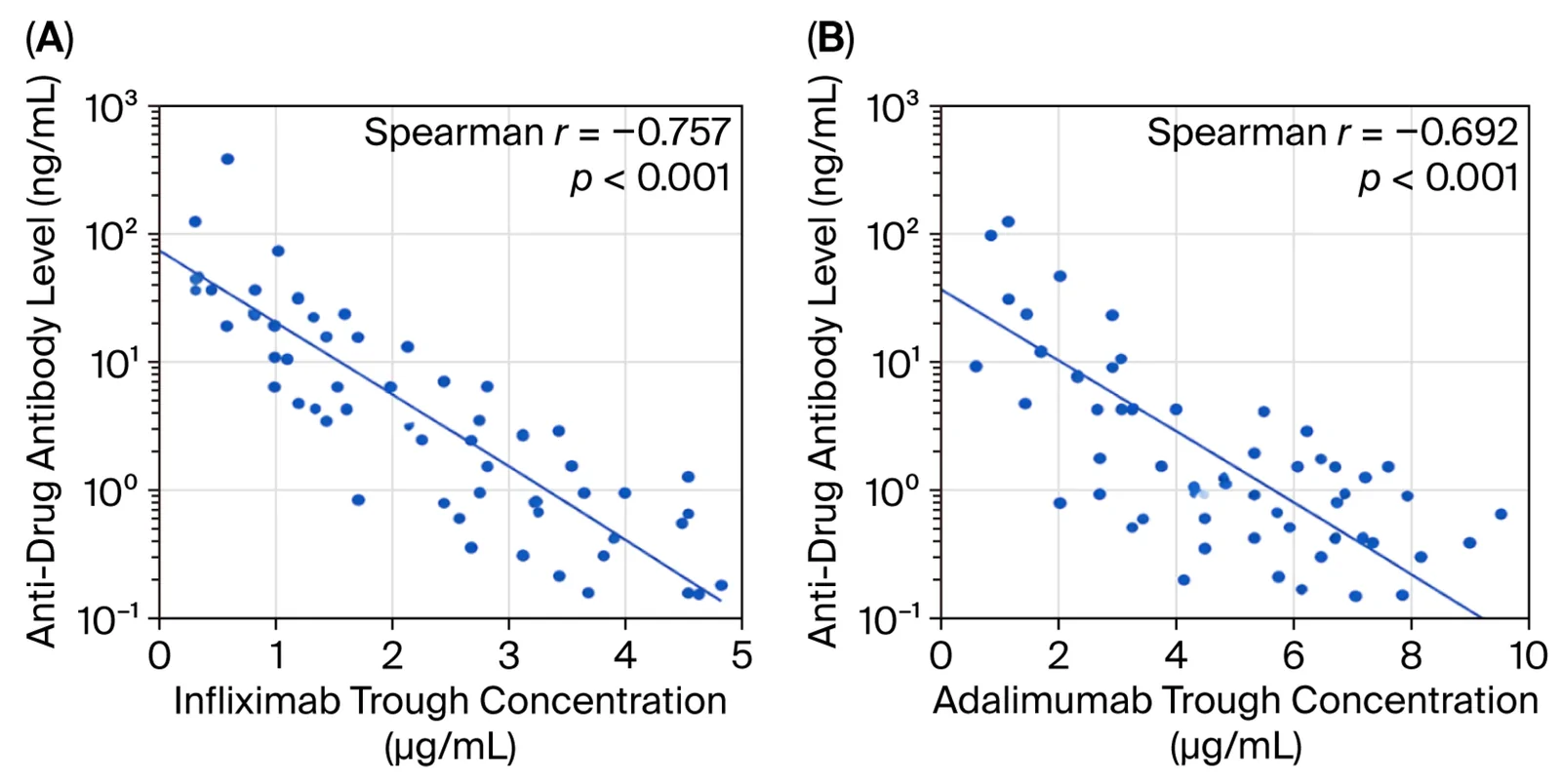
(**A**) Scatterplot showing the inverse association between infliximab trough concentrations and anti-drug antibody (ADAb) levels. (**B**) Scatterplot showing the inverse association between adalimumab trough concentrations and anti-drug antibody (ADAb) levels. ADAb levels are displayed on a logarithmic scale. Note: Each point represents an individual patient. The solid line represents the fitted regression line.

Among infliximab-treated patients, trough concentrations demonstrated a strong inverse correlation with ADAb levels (r = −0.757, *p* < 0.001). Similarly, among adalimumab-treated patients, trough concentrations were inversely correlated with ADAb levels (r = −0.692, *p* < 0.001). These associations are illustrated in Figure 4A,B.

Sensitivity analyses performed after conservative winsorization of biologically extreme ADAb values demonstrated attenuation of correlation strength while preserving the inverse direction of association, supporting the robustness of the observed pharmacokinetic–immunogenic relationship.

### 3.7. Receiver Operating Characteristic (ROC) Analysis for ADAb Level as Non-Response Predictor

The ADAb cutoff value for predicting non-response in infliximab-treated patients was 0.02 ng/mL, with an area under the curve (AUC) of 0.624, sensitivity of 75%, and specificity of 47.6%. In adalimumab-treated patients, the ADAb cutoff value was 60 ng/mL, with an AUC of 0.689, sensitivity of 59.1%, and specificity of 76.5%. These results are shown in Supplementary Table S1.

### 3.8. Subgroup and Pharmacokinetic–Immunogenic Analyses

Subgroup analyses according to disease subtype demonstrated that significant differences in trough concentrations between responders and non-responders persisted in both Crohn’s disease and ulcerative colitis cohorts.

Combination therapy subgroup analyses based on therapeutic response also demonstrated significantly higher trough levels and lower ADAb concentrations among patients receiving concomitant azathioprine therapy compared with monotherapy (*p* < 0.001) for both biologics.

Furthermore, pharmacokinetic–immunogenic classification revealed that patients with subtherapeutic trough concentrations and elevated ADAb levels demonstrated the highest rates of therapeutic failure, whereas patients with therapeutic trough concentrations maintained high response rates regardless of ADAb status.

Because these analyses were exploratory and mechanistic in nature, detailed results are provided in the Supplementary Material (Supplementary Tables S2–S5).

## 4. Discussion

This cross-sectional study evaluated the pharmacokinetic profiles and clinical characteristics of Iraqi patients with inflammatory bowel disease receiving maintenance anti-TNF therapy (infliximab or adalimumab), providing crucial real-world evidence that validates the utility of therapeutic drug monitoring. The findings demonstrated that therapeutic response was associated with adequate serum anti-TNF trough concentrations, whereas elevated anti-drug antibody (ADAb) levels were linked to therapeutic failure and reduced biologic exposure. Furthermore, multivariable and subgroup analyses supported the importance of pharmacokinetic and immunogenic mechanisms in therapeutic outcomes across both Crohn’s disease and ulcerative colitis cohorts.

Anti-TNF therapy has substantially transformed the management of moderate-to-severe inflammatory bowel disease and remains a cornerstone of current treatment strategies [4,14,15]. Nevertheless, secondary loss of response remains one of the major limitations of long-term anti-TNF therapy and represents a frequent cause of dose escalation, biologic switching, hospitalization, and surgery [7]. In the present study, baseline demographic and clinical characteristics did not differ significantly between responders and non-responders, suggesting that therapeutic outcomes were more strongly influenced by pharmacokinetic and immunogenic factors than by baseline patient-related characteristics. These findings are consistent with previous therapeutic drug monitoring studies demonstrating that inter-individual variability in biologic clearance and immunogenicity substantially contributes to therapeutic heterogeneity among anti-TNF-treated patients [24,25].

Exceptions among baseline characteristics that differed significantly between responders and non-responders were limited to the disease activity indices. Therapeutic response in this cohort was defined by clinical assessment using validated disease activity indices: HBI for Crohn’s disease and SCCAI for ulcerative colitis, with established thresholds distinguishing clinical remission in responders versus non-responders. These clinical classifications were supported by CRP, a sensitive systemic inflammatory marker. From a clinical standpoint, these findings emphasize the importance of combining disease activity scores with an objective biomarker like CRP [18,19].

One of the principal findings of the present study was the strong association between serum trough concentrations and therapeutic response. Responders receiving either infliximab or adalimumab had significantly higher trough concentrations compared with non-responders. Moreover, ROC curve analysis demonstrated strong discriminative performance of serum trough concentrations for predicting therapeutic response, with optimal cutoffs of 3.0 µg/mL for infliximab and 4.98 µg/mL for adalimumab. These findings are highly consistent with current therapeutic drug monitoring recommendations and previously proposed pharmacokinetic targets [10,20,21,22,23]. Previous studies have similarly demonstrated that maintenance trough concentrations above approximately 3–5 µg/mL for infliximab and 5–7.5 µg/mL for adalimumab are associated with sustained remission and improved long-term outcomes [11,21,23,26]. The high area under the curve values observed in our cohort further support the strong discriminative ability of serum drug exposure for predicting therapeutic success.

Multivariable logistic regression analysis additionally demonstrated that higher trough concentrations remained independently associated with therapeutic response after adjustment for clinically relevant covariates, further supporting the importance of adequate biologic exposure for optimizing anti-TNF therapy. These findings reinforce the concept that adequate biologic exposure is a key determinant of therapeutic efficacy and support the integration of pharmacokinetic-guided treatment optimization into routine clinical practice. Although the markedly elevated odds ratio observed for trough concentrations likely reflects partial separation and small-sample effects within the study cohort, the observed direction of association remains biologically plausible and consistent with contemporary pharmacokinetic evidence.

Immunogenicity represents another major contributor to anti-TNF treatment failure [27,28]. In the present study, non-responders demonstrated substantially higher ADAb levels than responders, particularly among adalimumab-treated patients. Furthermore, strong inverse correlations were observed between serum trough concentrations and ADAb levels for both infliximab and adalimumab, supporting the established pharmacokinetic relationship whereby antibody formation accelerates biologic clearance and reduces circulating drug exposure [11,28]. These findings are highly consistent with the landmark PANTS study, which identified immunogenicity as a major factor associated with anti-TNF treatment failure and reduced drug persistence [29].

Importantly, sensitivity analyses performed after conservative winsorization of biologically extreme ADAb values demonstrated attenuation of correlation strength while preserving the inverse direction of the association. This observation suggests that biologically extreme immunogenicity responses partially contributed to the magnitude of observed associations without altering the overall pharmacokinetic–immunogenic relationship. Additionally, ROC analysis demonstrated that ADAb levels had a fair ability to predict non-response in patients receiving adalimumab (AUC = 0.689), whereas predictive performance was lower in patients receiving infliximab (AUC = 0.624). Thus, the infliximab cutoff, which showed higher sensitivity but lower specificity, indicates better detection of non-responders, as opposed to adalimumab, which showed greater accuracy in identifying true responders, demonstrated by its higher specificity.

Subgroup analyses according to disease subtype further strengthened the robustness of our findings. Significant differences in trough concentrations between responders and non-responders persisted across both Crohn’s disease and ulcerative colitis cohorts. These observations support the applicability of pharmacokinetic-guided therapeutic optimization across different IBD subtypes and are consistent with previous studies that report similar exposure–response relationships in both diseases [11,26,27].

Another clinically important observation in the present study was the impact of concomitant azathioprine therapy. Although combination therapy was not independently associated with therapeutic response, patients receiving azathioprine demonstrated significantly lower ADAb concentrations compared with patients receiving anti-TNF monotherapy. These findings support previous evidence suggesting that immunomodulators suppress anti-TNF immunogenicity by reducing antibody formation [28,30,31]. Landmark studies such as the SONIC trial have previously demonstrated superior pharmacokinetic outcomes and lower immunogenicity rates with infliximab–azathioprine combination therapy compared with monotherapy [31]. However, the absence of a statistically significant improvement in clinical response rates among combination therapy recipients in our cohort may reflect the relatively limited sample size and cross-sectional design.

The combined pharmacokinetic–immunogenic classification model provided additional mechanistic insight into therapeutic failure patterns. Patients with subtherapeutic trough concentrations and elevated ADAb levels demonstrated the highest rates of therapeutic non-response, whereas patients with therapeutic trough concentrations maintained high response rates regardless of ADAb status. Few studies conducted during induction and maintenance phases reported the uncommon paradox in which patients continued to respond despite high ADAb levels, provided their trough drug levels remained adequate [32]. These findings closely mirror contemporary therapeutic drug monitoring algorithms that distinguish immune-mediated pharmacokinetic failure from non-immune pharmacokinetic failure and mechanistic failure [22,23]. Such classification frameworks may facilitate individualized treatment decisions, including dose intensification, addition of immunomodulators, switching within the anti-TNF class, or transitioning to biologic agents with alternative mechanisms of action [22,30,33].

Multiple confounding factors influence the response to anti-TNF therapy in patients with inflammatory bowel disease. These factors—commonly categorized as patient-related, disease-related, drug-related, genetic, immuno-serological, and miscellaneous predictors—are routinely considered when assessing biologic treatment effectiveness. Patient-related variables include age, sex, body mass index, and smoking status. Disease-related predictors encompass disease phenotype and duration. Drug-related determinants include serum trough concentrations, anti-drug antibody levels, and prior exposure to anti-TNF agents. Genetic markers (for example, HLA-DQA1*05) and serological markers such as C-reactive protein (CRP), fecal calprotectin, oncostatin M, and selected cytokines (including TNF-α) have also been investigated as predictors of response [34]. Combining multiple predictors of treatment response may provide a more robust and accurate prediction of therapeutic outcomes.

The present study was conducted at a single tertiary care center with a dedicated IBD service, which has important implications for potential selection bias. Patients in our cohort are referred to this center for specialized care and biologic therapy, often after failure or intolerance of conventional agents and, in some cases, prior biologics. Consequently, the clinical profile, prior treatment exposure, and selective use of TDM in patients with inadequate response or suspected loss of response in our cohort may not fully reflect the broader IBD population treated in community settings or in other healthcare systems. These center-specific referral patterns and practice characteristics could influence observed treatment responses, pharmacokinetic parameters, and immunogenicity rates, and may limit the generalizability of our findings to populations with different disease severity, ethnic and genetic backgrounds, or models of care.

This study has several limitations that should be acknowledged. First, the relatively modest sample size and single-center design may limit the generalizability of the findings to broader IBD populations. Second, the cross-sectional observational design precludes assessment of temporal relationships between ADAb formation, declines in serum trough concentrations, and subsequent clinical relapse; therefore, causal inferences cannot be established. Third, objective endoscopic assessment and fecal calprotectin measurements were not available, limiting comprehensive evaluation of mucosal healing and transmural inflammatory activity. Additionally, although highly significant pharmacokinetic associations were observed, the strong discriminative performance identified in ROC analyses should be interpreted cautiously because partial separation and cohort-specific effects may have influenced model performance. We recognize that performing multiple subgroup and ROC analyses increases the chance of type I error. Therefore, these analyses were considered exploratory and hypothesis-generating rather than confirmatory and must be interpreted with caution.

## 5. Conclusions

Therapeutic response among Iraqi patients with inflammatory bowel disease receiving infliximab or adalimumab therapy was associated with adequate serum trough concentrations and lower immunogenicity. Therapeutic drug monitoring demonstrated substantial clinical utility in identifying pharmacokinetic and immune-mediated mechanisms associated with treatment failure; these differences were evident between responders and non-responders and across subgroups (Crohn’s disease vs. ulcerative colitis; monotherapy vs. combination therapy). Integration of reactive TDM into routine clinical practice may facilitate individualized treatment optimization and improve long-term therapeutic outcomes in patients receiving anti-TNF therapy. Larger prospective multicenter studies incorporating objective inflammatory biomarkers and endoscopic outcomes are warranted to validate these findings.

## Figures and Tables

**Table 1:** Patients’ characteristics and demographics by therapeutic outcomes.

Variable	Responders (*n* = 38)	Non-Responders (*n* = 42)	*p*-Value
Age (years)	26.5 (22–40.75)	30 (24–40.25)	0.472 ^a^
Disease duration (months)	60 (36–84)	48 (27–81)	0.621 ^a^
BMI (Kg/m^2^)	25.1 (±4.6)	24.42 (±5.81)	0.559 ^b^
Age at diagnosis (years)	22.5 (17–33)	24 (19.25–31.75)	0.524 ^b^
Gender			
Male	21 (55.3%)	21 (50%)	0.661
Female	17 (44.7%)	21 (50%)	
Disease type			
CD	26 (68.4%)	24 (57.1%)	0.358
UC	12 (31.6%)	18 (42.9%)	
Anti-TNF Type			
Infliximab	21 (55.3%)	20 (47.6%)	0.512
Adalimumab	17 (44.7%)	22 (52.4%)	
Azathioprine use			
Users	20 (52.6%)	15 (35.7%)	0.176
Non-users	18 (47.4%)	27 (64.3%)	
Dosing strategy			
Standard dosing	29 (76.3%)	26 (61.9%)	0.228
Intensified dosing	9 (23.7%)	16 (38.1%)	
Anti-TNF use			
Previous users	10 (26.3%)	5 (11.9%)	0.151
First users	28 (73.7%)	37 (88.1%)	
Steroid Use			
Yes	0 (0%)	4 (9.5%)	0.117
No	38 (100%)	38 (90.5%)	
Family History of IBD			
Positive	5 (13.2%)	5 (11.9%)	1.00
negative	33 (86.8%)	37 (88.1%)	
History of Intestinal surgery			
Positive	6 (15.8%)	8 (19%)	0.774
negative	32 (84.2%)	34 (81%)	
Smoking status			
Non-smokers	28 (73.6%)	29 (69%)	0.186
Ex-smokers	5 (13.2%)	2 (4.8%)	
Current smokers	5 (13.2%)	11 (26.2%)	
Therapy duration			
<12 months	14 (36.8%)	15 (35.7%)	0.857
(12–24) months	8 (21.1%)	11 (26.2%)	
>24 months	16 (42.1%)	16 (38.1%)	
Disease Activity Index			
HBI (for CD patients)	2.5 (1–3)	6 (5–9.25)	<0.001
SCCAI (for UC patients)	1 (0.25–1.75)	4 (3.75–5.25)	<0.001

Values are presented as median (interquartile range), mean ± standard deviation, or number (%), as appropriate. BMI: body mass index; ^a^ Mann–Whitney U test; ^b^ Independent-samples *t*-test. Categorical variables were analyzed using a Chi-square test or Fisher’s exact test, as appropriate.

**Table 2:** Multivariate logistic regression analysis of infliximab-treated patients.

Predictor	Adjusted Odds Ratio	*p*-Value
Trough level (µg/mL)	2.57	0.01 *
ADAb level (ng/mL)	0.09	0.28
Azathioprine use (users vs. non-users)	0.4	0.203
Anti-TNF use (previous vs. first users)	0.3	0.471
Disease type (CD/UC)	2.04	0.352

* Significance, *p* < 0.05.

**Table 3:** Multivariate logistic regression analysis of adalimumab-treated patients.

Predictor	Adjusted Odds Ratio	*p*-Value
Trough level (µg/mL)	2.05	0.03 *
ADAb level (ng/mL)	0.038	0.992
Azathioprine use (users vs. non-users)	1.04	0.947
Anti-TNF use (previous vs. first users)	0.4	0.229
Disease type (CD/UC)	0.852	0.824

* Significance, *p* < 0.05.

**Table 4:** Association between anti-drug antibody levels and therapeutic response.

Drug	Therapeutic Response	*n*	ADAb Level (ng/mL)	*p*-Value
Infliximab	Responders	21	0.01 (0.01–0.02)	0.178 ^a^
	Non-responders	20	0.02 (0.01–0.05)	
Adalimumab	Responders	17	48.3 (35.7–59.7)	0.047 ^a^
	Non-responders	22	61.35 (48.75–84.83)	

Values are presented as median and interquartile range (IQR); ^a^ Mann–Whitney U test.

## Data Availability

The data presented in this study are available from the corresponding author upon request due to restrictions (e.g., privacy, legal or ethical reasons).

## Supplementary Materials

The following supporting information can be downloaded at https://www.mdpi.com/article/10.3390/diseases14090325/s1, Table S1. ROC Analysis for ADAb level Predicting Non-response. Table S2. Subgroup Analysis by Disease Type: Trough and ADAb Levels According to Therapeutic Response. Table S3. Infliximab Combination Therapy Analysis. Table S4. Adalimumab Combination Therapy Analysis. Table S5. Combined pharmacokinetic-immunogenic model .

## Abbreviations

The following abbreviations are used in this manuscript:

TDM	Therapeutic drug monitoring
Anti-TNF	Anti-tumor necrosis factor
IBD	Inflammatory bowel disease
ADAb	Anti-drug antibody
ELISA	Enzyme-linked immunosorbent assay
CD	Crohn’s disease
UC	Ulcerative colitis
IFX	Infliximab
ADM	Adalimumab
PNR	Primary non-response
LOR	Loss of response
STROBE	Strengthening the Reporting of Observational Studies in Epidemiology
BMI	Body mass index
HBI	Harvey–Bradshaw Index
SCCAI	Simple Clinical Colitis Activity Index
CRP	C-reactive protein
TNF-α	Tumor necrosis factor-alpha
HLA-DQA1*05	Human leukocyte antigen-DQA1*05
