# Supplementary material for: Associations Between Anti-TNF Pharmacokinetics, Immunogenicity, and Therapeutic Response in Iraqi Patients with Inflammatory Bowel Disease: A Real-World Therapeutic Drug Monitoring Study
Source: Diseases. 2026 Sep 5;14(9):325. doi: 10.3390/diseases14090325 (PMC13605217; doi:10.3390/diseases14090325)
Supplement: Supplementary file 1 [file diseases-14-00325-s001.zip › diseases-4399753-supplementary.pdf]

IBD-Diseases 4399753 / Supplementary Materials

Table S1. ROC Analysis for ADAb level Predicting Non-response

Drug	ADAb cutoff	AUC	Sensitivity	Specificity
Infliximab	0.02 ng/mL	0.624	75.0%	47.6%
Adalimumab	60.00 ng/mL	0.689	59.1%	76.5%

Table S2. Subgroup Analysis by Disease Type: Trough and ADAb Levels According to Therapeutic Response

Disease Type	Drug	Marker	Responders n	Responders Median (IQR)	Non-responders n	Non-responders Median (IQR)	P value
Crohn's disease	Infliximab	Trough level (µg/mL)	17	3.65 (3.40-4.10)	12	0.96 (0.89-1.06)	**<0.001***
		ADAb level (ng/mL)	17	0.01 (0.01-0.02)	12	0.02 (0.01-0.02)	0.450
Crohn's disease	Adalimumab	Trough level (µg/mL)	9	5.82 (5.67-7.15)	12	3.65 (3.04-4.81)	**<0.001***
		ADAb level (ng/mL)	9	47.40 (38.70-56.70)	12	61.35 (50.85-72.22)	0.070
Ulcerative colitis	Infliximab	Trough level (µg/mL)	4	4.25 (3.99-4.45)	8	1.10 (1.09-2.02)	**0.008***
		ADAb level (ng/mL)	4	0.05 (0.02-3.21)	8	0.03 (0.02-10.08)	1.000
Ulcerative colitis	Adalimumab	Trough level (µg/mL)	8	6.59 (5.77–7.12)	10	2.71 (2.60-3.47)	**<0.001***
		ADAb level (ng/mL)	8	51.90 (35.62–62.92)	10	72.75 (46.35-139.57)	0.315

***Significant, P <0.05**

Table S3. Infliximab Combination Therapy Analysis

Marker	Azathioprine users (n=22) (Responders/non-responders)	P value	Azathioprine non-users (n=19) (Responders/non-responders)	P value
Trough level (ug/mL)	3.9 (3.4-4.32) / 0.95 (0.81-1.08)	**<0.001**[a]	3.65 (3.35-4.1) / 1.1 (0.975-1.27)	**<0.001**[a]
ADAb level (ng/mL)	0.01 (0.01-0.02) / 0.5 (0.21-1.05)	**<0.001**[a]	0.5 (0.7-3.7) / 1.77 (1.16-5.26)	**<0.001**[a]
Response rate	14 (63.64%) / 8 (36.36%)		7 (36.84%) / 12 (63.16%)	

[a]Mann-Whitney U test, ***Significant, P <0.05**

Table S4. Adalimumab Combination Therapy Analysis

Marker	Azathioprine users (n=13) (Responders/non-responders)	P value	Azathioprine non-users (n=26) (Responders/non-responders)	P value
Trough level (ug/mL)	6.07 (5.43-7.2) / 3.56 (2.99-4.92)	**<0.001**[a]	6.49 (5.4-7.61) / 2.97 (2.6-4.44)	**<0.001**[a]
ADAb level (ng/mL)	25.7 (18.3-29.7) / 62.7 (57.7-67.4)	**<0.001**[a]	32.15 (19.73-42.07)/ 89.6 (76.2-92.51)	**<0.001**[a]
Response rate	6 (46.15%) / 7 (63.85%)		11 (42.31%) / 15 (57.69%)	

[a]Mann-Whitney U test, ***Significant, P <0.05**

Table S5. Combined pharmacokinetic-immunogenic model

Group	N	Responders	Non-responders
Subtherapeutic trough / High ADAb	22	0 (0.0%)	22 (100.0%)
Subtherapeutic trough / Low ADAb	22	3 (13.6%)	19 (86.4%)
Therapeutic trough / High ADAb	11	11 (100.0%)	0 (0.0%)
Therapeutic trough / Low ADAb	25	24 (96.0%)	1 (4.0%)
